# Development of a Child-Informed Measure of Subjective Well-Being for Research on Residential Care Institutions and Their Alternatives in Low- and Middle-Income Countries

**DOI:** 10.1007/s10560-024-00968-x

**Published:** 2024-04-14

**Authors:** Sarah Elizabeth Neville, Joanna Wakia, John Hembling, Beth Bradford, Indrani Saran, Margaret Lombe, Thomas M. Crea

**Affiliations:** 1 Boston College School of Social Work, Chestnut Hill, MA USA; 2 Maestral International, Minneapolis, MN USA; 3 Catholic Relief Services, Baltimore, MD USA; 4 Boston University School of Social Work, Boston, MA USA

**Keywords:** Alternative care, Child participation, Non-parental care, Child welfare, Child Protection

## Abstract

10% of children worldwide live in households without a biological parent, and 5.4 million children live in residential care institutions. This study describes a participatory, child-informed process of developing a multidimensional measure of child subjective well-being tailored towards the priorities of children who have lived in residential care. Eight focus groups were held with *n* = 49 adolescents reunified with family after living in residential care in Kenya and Guatemala and six focus groups were held with *n =* 29 young adults who had lived in residential care during childhood. After analysis of the focus groups, and using the Orphans and Vulnerable Children Wellbeing Tool as a foundation, the resulting tool contained 43 survey questions. Member checking, translation, and cognitive interviewing were conducted. The survey was administered to *N =* 180 young people in Kenya and Guatemala who were reunified with family after living in residential care or at risk of entering residential care. Exploratory factor analysis indicated that the measure had three salient factors with good convergent validity and internal consistency: *care and safety* (12 items), *basic needs* (13 items), and *leisure and freedom* (7 items). This study contributes a new, psychometrically validated survey measure that can be used to assess the well-being of children connected to residential care, as well as a replicable model for creating contextualized quantitative measures via child participation that can inform policymaking on children’s care in low- and middle-income countries.

In its landmark adoption of the Guidelines for the Alternative Care of Children, the United Nations asserted that “every child and young person should live in a supportive, protective and caring environment that promotes his/her full potential,” and that “children with inadequate or no parental care are at special risk of being denied such a nurturing environment” (United Nations General Assembly, 2010, para. 4). Globally, one in ten children lives in a household without their biological parents (Martin & Zulaika, 2016), and an estimated 5.4 million children are living in residential care institutions (RCIs), also called children’s homes or orphanages (Desmond et al., 2020). Human rights and child rights advocates have underscored the importance of children who live outside of parental care (i.e., in “alternative care”) having an active role in any policymaking about vulnerable children’s care and protection (United Nations General Assembly, 2010). In tandem, researchers have been using quantitative methods for decades to examine the well-being of children who live in or formerly lived in residential care (van IJzendoorn et al., 2020). In general, such investigations have aimed to determine the causal impact of living in residential care institutions and of family reintegration on children’s well-being, often by comparing children living in residential care and children living in other settings. The findings of these studies have been used to make policy recommendations regarding what form of alternative care is most suitable for children for whom it is not in their best interests to live in parental care. This evidence is also driving the global efforts of care reform to favor alternative family-based care over residential care, and to drive “deinstitutionalization” of child welfare systems so that children in institutions can be returned to family-based care (Goldman et al., 2020).

However, in studies of children who have lived in residential care, some child well-being outcomes receive more attention than others. European children in infancy and early childhood are overrepresented compared to older children and those from other regions (van IJzendoorn et al., 2020; Whetten et al., 2009). Thus, the most common well-being outcome measures employed are relevant to children’s earliest years of life, which may not be relevant to older children, and are designed for a Western context, which may not be relevant for many low- and middle-income countries. This is well-illustrated by the 2020 Lancet Commission on the Institutionalisation and Deinstitutionalisation of Children, the purpose of which was to conduct a systematic literature review to answer the question of whether growing up in residential care institutions negatively affects development or mental health, and whether leaving institutions and joining families leads to recovery from these adverse trajectories (van IJzendoorn et al., 2020). Their database search strategy was designed to include a wide range of well-being outcomes, including medical-related ones (e.g., growth, stress regulation, respiratory-related, nutrition), as well as education, delinquency, and others. Ultimately, within the 308 studies they included in their meta-analysis, 55 studies measured child physical growth, 46 measured physical health, 20 head circumference (as a proxy for brain development), 116 cognition, 146 socioemotional development, and 28 attention. Although such researcher-driven, objective measurements of child well-being can and do lend to important insights, children’s voices and perspectives on residential care and family reintegration are also crucial to understanding outcomes and respecting child rights within the context of policymaking and research on vulnerable children (United Nations General Assembly, 2010). However, of the outcomes included in the Lancet Commission, only “socioemotional development” could potentially measure children’s own perspectives, and it is unclear what measures were used in the studies included in this category. Thus, there is clearly more work to be done to include children’s self-reports on their well-being into this body of quantitative research.

## The Quantitative Measurement of Well-Being

“Well-being” is a concept that typically aims to capture a comprehensive range of positive life outcomes. Well-being can be conceptualized as objective and subjective: objective well-being refers to observable indicators of life quality (e.g., household income, illness diagnosis, educational attainment), while subjective well-being is based only on an individual’s perspective of their own life (sometimes also referred to as happiness or life satisfaction) (Voukelatou et al., 2021). An individual’s objective and subjective well-being may not be the same: for example, a child may be “objectively” assessed as having low well-being if they have health problems or live in poverty, but subjectively state they are satisfied with their life. Similarly, an “objective” metric might determine a child is enjoying well-being if their nutritional and educational needs are being met, even if, when asked, the child reports being unhappy with their living situation.

Objective and subjective well-being can be measured unidimensionally or multidimensionally. For example, annual household income is a common unidimensional indicator of objective well-being. Unidimensional indicators of subjective well-being include, for example, answers to questions like, “Overall, how satisfied are you with your life right now?” or responses from the “Cantril Ladder” tool, wherein respondents imagine a ladder where the top represents the best possible life and the bottom the worst, and select a step of the latter to represent their own quality of life (OECD, 2013). Others recognize the importance of using multidimensional measures, that is, evaluating multiple dimensions or aspects of well-being. For example, the OECD suggests evaluating adult well-being with a variety of objective measures related to income, wealth, housing, employment, education, health, and more (OECD, 2011). There are also multidimensional measures of subjective well-being, like Oxfam’s Humankind Index for Scotland, which asks participants to rate their own well-being in 18 sub-domains as varied as health, relationships, safety, leisure, and transportation (Oxfam Scotland, 2013; Walker et al., 2012).

Conceptualizations of well-being can also differ by context and population. Organizations including the World Health Organization and Oxfam have recognized this, and as a result, have used qualitative methods, including focus groups and ranking exercises, to determine which dimensions of well-being are important to specific populations, such as residents of Scotland or community members in coastal Kenya (McGregor et al., 2015; Walker et al., 2012; World Health Organization, 1998). They have used these findings to create quantitative, multidimensional well-being measures to inform policymaking and practice that affect these populations (McGregor et al., 2015; Walker et al., 2012; World Health Organization, 1998); surveys using these measures could, for example, identify subpopulations with lower well-being that need special attention in social programs, or determine which domains of well-being should be prioritized in government budgets (e.g., health programs versus housing assistance).

Children are one such population type that can also have unique conceptualizations of well-being, and so measures of well-being specific to children have been developed as well. For example, the Personal Well-being Index: School Children (PWI-SC) measures children’s subjective well-being in seven domains (standard of living, personal health, achievement in life, personal relationships, personal safety, feeling part of the community, and future security), using items like, “How happy are you about how safe you feel?” (Cummins & Lau, 2005). The scale is designed for use with any children (e.g., in high- or low-income countries, and who are not necessarily involved with child protection systems), and thus the questions are quite broad and non-specific; a validation study with Australian adolescents found it had good psychometric properties (Tomyn & Cummins, 2011). Children involved with child protective systems and out-of-home care are an even more specialized population whose conceptualizations of well-being may be unique. In England, researchers created a well-being measure specifically for English children living outside of parental care in order to more finely assess their needs and adjust child welfare policies accordingly (Selwyn et al., 2017; Wood & Selwyn, 2017; Zhang & Selwyn, 2020). They first held focus groups with children in out-of-home (i.e., non-parental) care, then used the results to draft indicators, conducted member-checking with a subset of focus group participants, reduced the number of items to a manageable amount, piloted the survey, conducted cognitive interviewing, and finally revised the survey, resulting in an questions that when tested with English and Welsh children had overall good reliability and validity and an α of 0.80 (Selwyn et al., 2017; Wood & Selwyn, 2017; Zhang & Selwyn, 2020).

So while measures specific to children’s subjective well-being have been created, and even measures for children connected to child protective services in high-income countries, to our knowledge, no participatory or qualitative process of measure development has been conducted with children who have experienced residential care in low- and middle-income countries. One systematic review analyzed measures used to assess child outcomes in residential care (Wright et al., 2019). It found a few measures of child subjective well-being have been used in extant literature, including the WHOQOL-BREF (a measure developed with adults globally), used with children in Ghana (Salifu Yendork & Somhlaba, 2014), the Generic Children’s Quality of Life scale (developed with children in the UK), and the Children’s Happiness Scale (developed for children in non-parental care in the UK), both used with children in a 13-country study (Pandya, 2018). However, no measures of subjective well-being captured by this systematic review had been tailored for children with the experience of non-parental or residential care in low- and middle-income countries (in fact, only seven of the 38 studies were done in Africa and four in Latin America). The current study is designed to contribute to filling this gap in the literature.

## Study Aims and Context

The aim of this study was to create a multidimensional measure of subjective well-being informed by qualitative research with young people with lived experiences of residential care in two low- and middle-income countries. Ultimately we hope that such a tool can foster child participation in research and policymaking around the care of vulnerable children globally (United Nations General Assembly, 2010).

The data for this study were collected within the context of Changing the Way We Care (CTWWC), an initiative operating in multiple countries, including Kenya and Guatemala, to support the reform of child care systems, improve family strengthening support and alternative family-based care and reunify children in residential care with families where possible (Catholic Relief Services, n.d.). Kenya and Guatemala have different factors that affect children’s entry into alternative care, and residential care specifically, as well as a different type of system governing alternative care, yet similarities also exist across the two countries. Both countries have similar proportions of children living in residential care (Desmond et al., 2020), and family poverty and child maltreatment are reasons children enter residential care in both countries (Changing the Way We Care, 2020; Chege & Ucembe, 2020; Manzo Chávez, 2021). Drivers of entry into residential care more prominent in Guatemala than Kenya include malnutrition, community violence, and international migration, while issues more salient in Kenya include risk of female genital mutilation and early marriage (Changing the Way We Care, 2020; Chege & Ucembe, 2020; Kirk et al., 2017; Manzo Chávez, 2021). Children in Guatemala also usually enter residential care per order of the judicial system (Changing the Way We Care, 2020), while in Kenya the involvement of the judicial system is smaller (Kenya Department of Children’s Services, 2020).

Some international actors, including both researchers and policymakers, view the issue of residential care through a global lens, by drawing conclusions about and making recommendations for residential care that apply regardless of the continent, country, or region (United Nations General Assembly, 2010; van IJzendoorn et al., 2020; Whetten et al., 2014). Creating standardized data collection tools of subjective well-being that can be used across regions, yet are tailored for the specific context of children connected to residential care, will not only potentially aid in making some global generalizations about residential care for children, but also allow for comparative analyses that can examine nuances and differences between contexts. Moreover, a measure of subjective child well-being designed to apply to multiple regions can also be used as a basis for further adaptations in situations where there are not enough time or resources to construct an entirely new measure. For these reasons, this study utilized data from two distinct contexts, Kenya and Guatemala, to construct a measure that captures areas of well-being that are potentially uniquely salient for children who have lived in residential care across varying contexts in low- and middle-income countries.

## Method

This study used a multi-step iterative process to create a measure of well-being that captures salient domains of subjective well-being for children who have experienced residential care. First, focus groups were conducted with children and young people, and the data were analyzed. This informed construction of a measure of subjective well-being for children in residential care, which was built from the foundation of the Catholic Relief Services Orphans and Vulnerable Children Wellbeing Tool (Senefeld et al., 2011). Second, face validity of draft items was assessed via member check-in with focus group participants. Third, cognitive interviewing was conducted with *n =* 5 children, before the survey was deployed amongst *N =* 180 children ages 11 to 18 who had lived in residential care in Kenya and Guatemala. Finally, we used exploratory factor analysis to determine the factor structure of the scale and create subscales. These procedures, which received ethical approval from the Boston College Institutional Review Board and the Maseno University Ethics Review Committee, are described below.

### Development of the Measure

#### Focus Group Procedure

Maximum variation sampling was used to design the focus group sampling strategy and recruitment targets (Creswell & Poth, 2018). In Kenya, four focus groups were conducted in each of the three study counties, Kisumu, Nyamira, and Kilifi. Six focus groups were held with *n =* 41 children ages 11 to 17 who had reunified with family after living in residential care with post-placement support from CTWWC. These children were sampled via stratified random sampling from CTWWC’s roster of children they supported, using strata for county and age (Table 1). Six focus groups were held with *n =* 29 young adults ages 18 to 29 who had lived in residential care during their childhoods more than two years ago. These young people were selected via convenience sampling from networks of young people who had lived in residential care with whom CTWWC had prior connections. In Guatemala, CTWWC served a smaller number of children, many of whom are younger than 11, and did not have existing networks of young adults, so convenience sampling was used to recruit *n =* 8 children ages 11 to 17 who had been reunified with families into two focus groups in two locations served by CTWWC, with five children in the Zacapa focus group and three in the Guatemala City focus group. All participants were invited via phone (in the case of children, their caregivers were contacted first). We intentionally sampled young people who had lived in multiple settings—who had lived in residential care, who had been reunified with family, and who had “aged out” of residential care during emerging adulthood—in hopes that our measure could ultimately be useful for comparing residential care with other forms of care.

**Table 1:** Sampling strata for focus groups

Kisumu County, Kenya				Nyamira County, Kenya				Kilifi County, Kenya				Guatemala	
Reunified adolescents		Young adults		Reunified adolescents		Young adults		Reunified adolescents		Young adults		Guatemala City group (*n* = 3)	Zacapa group (*n* = 5)
Younger group (*n =* 8)	Older group (*n =* 8)	Men’s group (*n =* 8)	Women’s group (*n =* 6)	Younger group (*n =* 3)	Older group (*n =* 5)	Men’s group (*n =* 3)	Women’s group (*n =* 3)	Younger group (*n =* 6)	Older group (*n =* 6)	Men’s group (*n =* 7)	Women’s group (*n =* 7)		

In the focus groups, trained and experienced Guatemalan and Kenyan facilitators who did not have prior relationships with the participants asked participants to reflect on and share their personal experiences of life in residential care and life after residential care. After this, participants were asked “Use your imagination to make up a child who lives in a residential care institution and is really, really happy—as happy as they could possibly be. What is their life like?” Facilitators were given probes to use if children were stuck on just a few topics; specifically, they were trained to ask about safety, health, food, housing, relationships, emotions and feelings, education, or livelihoods (Betancourt et al., 2010). They were also trained to probe about different types of children (e.g., boys and girls, children with disabilities). Then they asked, “Now use your imagination to make up a child who left a residential care institution and joined a family, who is really, really happy—as happy as they could possibly be. What is their life like?” Probing questions were the same, and in addition they could also ask about children who joined different types of families (e.g., grandparents, foster families).

Finally, facilitators labeled two flip charts with the following headings: “What is important for ‘doing well’ for children in residential care institutions” and “What is important for ‘doing well’ for young people in the first five years after leaving residential care”. Participants were asked to write what was important for ‘doing well’ on sticky notes or cards which they then pasted onto each flip chart. This written free-listing process allowed participants to distill the information from their previous discussion into a list, potentially incorporating not just their own ideas but also the ideas raised by fellow participants that resonated with them.

Participants’ only incentives were refreshments and transportation. Facilitators were trained on safeguarding procedures related to vulnerable children and adults, and all adult participants gave informed consent, while children gave assent and their parents gave informed consent. The focus groups were conducted in the summer of 2021. Focus group facilitators audio recorded the groups and transcribed the recordings. In Kenya, the facilitators also translated the audio recording into English where necessary (a mix of languages could be used in the groups), while in Guatemala, the transcriptions were fully in Spanish (since the lead researcher could read Spanish, they were not translated into English).

### Analysis of Focus Group Data

The lead researcher, a white American doctoral student with expertise in global alternative care, conducted a pragmatic, rapid analysis of the focus group transcripts and written lists to have results ready for programmatic use in a timely way. The process was entirely inductive, following grounded theory’s process of relying on participants’ voices to create a framework of well-being rather than using theories held *a priori* by the researchers (Creswell & Poth, 2018). After reading the transcripts, the lead researcher highlighted everything a participant mentioned as a sign of a good life or important to doing well. Each of these excerpts, whether it came from the discussion or from the participants’ written lists, constituted in vivo codes which were entered individually into a spreadsheet that also noted the context of the excerpt (Saldaña, 2016). The codes and excerpts were in English for the Kenya data, and in Spanish for the Guatemala data. Across the 12 Kenya focus groups, there were 909 excerpts of areas of well-being mentioned by participants, and 170 excerpts came from the Guatemala focus groups.

After finalizing the spreadsheet, the researcher considered the excerpts in their entirety and began to code them, according to common categories and themes. In some cases, once the researcher established a code, it stayed the same throughout the entire analysis (for example, the code “food”). In other cases, as it became clear that some categories were too narrow, or were connected to other categories, categories were changed, renamed, or split. For example, “community acceptance” was eventually merged with “sense of belonging” to be “acceptance/belonging”; on the other hand, while “hygiene” originally encompassed many aspects of sanitation, it became clear that “having sanitary towels” necessitated its own category. There were 42 themes that had more than two excerpts in Kenya, and 18 themes that had more than three excerpts in Guatemala; though not exhaustive, Tables 2 and 3 illustrate the most common themes from these data.

**Table 2:** Ten most frequently mentioned themes from Kenya focus groups

Code	Freq.	Example excerpts	Notes
Love/care	86	“He or she will be happy when surrounded by people who show them love”; “There is that love of a family”	Related to love, care, affection, “parental love”
Food	63	“balanced food, good food, changing menus”; “chakula chenya anukuta [sweet food finds me]”; “balanced diet”	
Guidance and counseling	58	“Having mentors for guidance and counseling”; “there are people who can render us pieces of advice…when you are emotionally troubled”; “boy has good relations with father and is guided on the roles of a man”	Participants often mentioned “guidance and counseling” verbatim; this referred to advice from adults
Security	52	“feel safe because they live in a secured compound”; “Protection from people who might not have good intentions with them”; “there should be having good security in the area”	Safety was often introduced by facilitators with probing questions. Participants often understood it as having a watchman or a gate.
Clothes	47	“Has care and basic needs, not going to school with hand stitched cloth and bare foot”; “inner pants and bikers”; “shoes”	
Education	44	“Being taken to school, being taught some skills”; “Provision of all educational requirement for each and every child”	
Health	41	“is taken to hospital for medication”; “are taken to the hospital when they fall sick”	
Being with/having family	39	“girl gets to always go places together with her mother”; “meeting the family after a long time”; “emotional bond with family”	Participants sometimes equated happiness as simply being with or having family.
Play	34	“The child should get enough playing time”; “don’t overwork, they have leisure time”; “can climb fruit trees and play with fellow children”	
Hygiene	30	“proper hygiene”; “proper sanitation”; “has washing soap”	Excluding sanitary towels, which had its own category

**Table 3:** Ten most frequently mentioned themes from Guatemala focus groups

Code	Freq.	Example excerpts	Notes
Good behavior	35	“Ser obediente” *(being obedient); “*Hacerles caso a los abuelitos” *(obey your grandparents); “*No estar mucho tiempo en la calle” *(not spending much time on the street); “*Ayudar a barrer si mama está cansada” *(helping your mom sweep if she is tired)*	Focus group facilitators believed that children mentioned this because if they do not listen, they are punished.
Play	35	“Jugar pelota” *(playing ball*); “columpios” *(swings);* “jugar shuco” *(playing freeze tag);* “ayudándole a papa a pescar” *(helping dad to fish)*	
Harmony and relationships	18	“Amor, Ayuda, Paciencia, Cariño, Sabiduria” *(love, help, patience, care, wisdom)*	This code was used for abstract items that related to positive relationships
Freedom	16	“No estar encerrados” *(not being locked in);* “puede salir” *(can go out)*	
Positive family relationships	13	“Que su familia lo apoyen” *(their family supports them);* “tiene mucho amor, cariño, comprensión” *(they have a lot of love, care, and understanding)*	
Being with family	11	“siempre tiene a su familia con ella” *(always has her family with her);* “Acompañar a la mamá a comprar” *(going shopping with your mom)*	
Food and nutrition	7	“tiene comida” *(has food);* “sus tres tiempos de comida” *(their three meals a day)*	
Education	8	“Graduarse” *(graduating from high school)*	
Shelter	8	“tendrá su cuarto aparte” *(has her own room);* “pila para bañarse” *(outdoor sink to bathe/wash in)*	
Clothing	8	“zapatos” *(shoes)*	

### Item Development

The researcher then compared the codes and ideas from the focus groups with a pre-identified well-being tool, the Catholic Relief Services Orphans and Vulnerable Children Wellbeing Tool (OWT) (Senefeld et al., 2011). The OWT is a self-report measure of well-being for children ages 13 to 18 who may be associated with orphan and vulnerable children (OVC) programs. OVC programs are targeted at children, adolescents, and young people living with or affected by HIV and AIDS. Children captured within the “OVC” category may or may not have involvement in RCIs, and children in RCIs may or may not be “OVC,” but the populations overlap and have some sociologically similar characteristics (e.g., with regards to stigma, poverty) and are sometimes conflated (Cheney & Rotabi, 2014). Thus, the research team found the OWT to be a relevant measure from which to build.

We modified existing questions and added questions to the tool until we had a final list of survey questions that encompassed the key themes from the children and young adults, while also being general enough to use as survey questions for all children who have lived in RCIs. Some domains of well-being arose from the data that the OWT did not contain, particularly around play and leisure as well as freedom to go out and personal agency, while others from the OWT remained relevant, such as *“I eat at least two meals a day”*. Some OWT items were dropped because they were not mentioned in the focus groups (e.g., *“My belief in God gives me strength to face difficulties”).* Several were changed; for example, one OWT item was *“My school attendance is affected by my need to work,”* but because some participants in Kenya noted that it was important for children to have adequate time to study at home (not just to attend class), the statement was broadened to, *“My work or chores impact my ability to do well in school”*. We also retained the OWT’s three-point Likert scale response, where respondents could respond whether statements were true for them all of the time, some of the time, or none of the time, which the OWT had adopted because it was simple enough for child respondents.

### Member Checking

Member checking is the process of verifying results and interpretation of research with research participants or members of the population being studied (Creswell & Poth, 2018). It is considered a best practice in qualitative and participatory research in particular, and has been utilized as a way of assessing face validity in similar studies that used qualitative data from marginalized groups to create contextually-relevant measures (Ng et al., 2014; Selwyn et al., 2017; Sharma et al., 2013).

In Kenya, facilitators of the original focus groups conducted member checking workshops in August 2021 with a convenience sub-sample drawn from the participants of the original six young adult and three older children focus groups, excluding all *n* = 19 participants of the three younger children groups (as they may not have been able to understand the abstract nature of the discussion); *n =* 22 adolescents and young adults participated in the three member-checking workshops (mean age = 18.1 years). Facilitators told participants they were providing them with a list of “the most important things to look at in order to determine if a child who currently lives in a residential care institution, or who used to live in a residential care institution, is doing well and having a good life…” Participants were asked to consider the lists of items, and suggest revisions, additions, or deletions. Facilitators took contemporaneous notes in English, which the lead researcher analyzed, and incorporated the respondents’ suggestions.

Participants largely noted that the list of items resonated with them. The changes that were made based on the member checking process were few; for example, participants suggested combining “I have a house where I can sleep at night” and “Where I sleep at night is comfortable,” so the revised version of the tool consolidated these items into, “I have a comfortable place to sleep at night”.

In Guatemala, as previously described, it was only possible to hold two in-person focus groups due to the low number of adolescents receiving post-placement services from CTWWC and because these adolescents were spread across various geographic regions. To supplement the focus groups, the facilitators conducted phone calls with *n =* 5 eighteen-year-olds who were reunified with family and receiving post-placement services from CTWWC. The team decided that the abstract nature of the discussion and the phone call format would be challenging for younger children. The phone calls in Guatemala served as both a way to expand the participant pool and collect new data, as well as to conduct member checking. Like the focus groups, facilitators first asked participants what life looks like for a child in residential care enjoying well-being, then the same regarding reunified children. Next, facilitators listed preliminary themes from the Guatemala focus groups, and asked participants what they thought about the list, and if they had any changes or additions. The facilitators audio recorded and transcribed these phone calls. Generally, the phone call participants generally did not disagree with anything the focus group participants said. Because the phone calls served the additional purpose of extending the focus group data collection (asking some of the same questions with new participants), the lead researcher analyzed the phone call data alongside the Guatemala focus group data as if they were focus groups.

#### Translation and Cognitive Interviewing

After completing member checking, and before administering the tool with children in Kenya and Guatemala, we conducted translation and cognitive interviews. In Kenya, the English version of the items were used, but Kenyan nationals who were contracted to oversee survey deployment also pre-translated some key terms and phrases into Ekegusii, Dholuo, and Kiswahili for survey enumerators to use if the respondent did not understand the terms in English. These key terms were not back-translated into English. In Guatemala, Guatemalan nationals contracted to oversee survey deployment translated the full measure into Spanish; there was no back-translation to English because multiple researchers were bilingual in English and Spanish and able to review the translation directly.

Cognitive interviewing (Collins, 2003) was conducted in person with *n =* 3 child respondents in Kenya (one from each study county) and *n* = 2 in Guatemala to assess whether children between ages 11 and 18 would understand and feel comfortable responding to the questions. The most substantive change that came as a result of cognitive interviewing was changing two items (“I’m treated differently from the other children in my household” and “I’m treated differently from other children in my village/neighborhood/compound/community”) in the Spanish version; cognitive interviewers found that the negative wording was confusing to respondents, and advised revising them into a positive framing (i.e., “I’m treated the same as…”). Thus, while these two items were reversely coded for Kenya respondents, they were not reverse coded for Guatemala respondents. The items that resulted from this process (see Table 4) were used to conduct further psychometric testing.

**Table 4:** List of items used in exploratory factor analysis and missing data

	Number of missing responses		
Item text	Kenya (*n =* 138)	Guatemala (*n =* 42)	Overall (*N =* 180)
At home, I have everything I need to keep myself clean	0 (0.0%)	0 (0.0%)	0 (0.0%)
I am happy with my clothing and shoes	0 (0.0%)	0 (0.0%)	0 (0.0%)
I have the materials I need for school	0 (0.0%)	1 (2.4%)	1 (0.6%)
I like my teachers at school	0 (0.0%)	1 (2.4%)	1 (0.6%)
My teachers treat me with respect	0 (0.0%)	1 (2.4%)	1 (0.6%)
My work or chores impact my ability to do well in school*	0 (0.0%)	1 (2.4%)	1 (0.6%)
I worry about having enough money for my education*	0 (0.0%)	0 (0.0%)	0 (0.0%)
I eat at least two meals a day	0 (0.0%)	0 (0.0%)	0 (0.0%)
I like the food I eat	0 (0.0%)	0 (0.0%)	0 (0.0%)
I can eat until I am satisfied	0 (0.0%)	0 (0.0%)	0 (0.0%)
My diet is well-balanced and nutritious	0 (0.0%)	2 (4.8%)	2 (1.1%)
My health is good	0 (0.0%)	0 (0.0%)	0 (0.0%)
I would be given medicine if I needed it	0 (0.0%)	0 (0.0%)	0 (0.0%)
Someone would take me to the hospital/clinic/doctor if I needed it	0 (0.0%)	0 (0.0%)	0 (0.0%)
If I needed something that my parents/caregivers can’t provide, there are others who would help	0 (0.0%)	0 (0.0%)	0 (0.0%)
I get to play and have fun	4 (2.9%)	0 (0.0%)	4 (2.2%)
I have enough time to study	0 (0.0%)	2 (4.8%)	2 (1.1%)
I have enough time to rest and sleep	1 (0.7%)	0 (0.0%)	1 (0.6%)
I get to pursue my hobbies and interests	0 (0.0%)	0 (0.0%)	0 (0.0%)
I have freedom to go out	1 (0.7%)	0 (0.0%)	1 (0.6%)
I have fun with my friends	1 (0.7%)	1 (2.4%)	2 (1.1%)
If I want something, my parents/caregivers will listen and consider it	0 (0.0%)	0 (0.0%)	0 (0.0%)
I can choose what to eat and when	0 (0.0%)	0 (0.0%)	0 (0.0%)
I am happy with how many friends I have	0 (0.0%)	0 (0.0%)	0 (0.0%)
I get along well with my friends	0 (0.0%)	3 (7.1%)	3 (1.7%)
I have someone to turn to for advice and guidance	0 (0.0%)	0 (0.0%)	0 (0.0%)
I have people I can talk to when I have a problem	0 (0.0%)	0 (0.0%)	0 (0.0%)
I have adults in my life who understand me	0 (0.0%)	0 (0.0%)	0 (0.0%)
The adults in my life teach me how to be successful in the future	1 (0.7%)	0 (0.0%)	1 (0.6%)
I feel I am supported by my relatives	0 (0.0%)	0 (0.0%)	0 (0.0%)
I feel like I’m part of my family	0 (0.0%)	0 (0.0%)	0 (0.0%)
I get love and care from my parents/caregivers	1 (0.0%)	0 (0.0%)	1 (0.6%)
I’m treated differently from [Spanish: the same as] the other children in my household^†^	0 (0.0%)	0 (0.0%)	0 (0.0%)
I’m treated differently from [Spanish: the same as] other children in my village/neighborhood/compound/community^†^	0 (0.0%)	1 (2.4%)	1 (0.6%)
I am as happy as other kids my age	0 (0.0%)	0 (0.0%)	0 (0.0%)
I have a comfortable place to sleep at night	0 (0.0%)	0 (0.0%)	0 (0.0%)
My home has a good environment for studying	0 (0.0%)	1 (2.4%)	1 (0.6%)
I feel safe where I live	0 (0.0%)	0 (0.0%)	0 (0.0%)
My home is peaceful	1 (0.0%)	0 (0.0%)	1 (0.6%)
I have someone to ask for help if I feel unsafe	0 (0.0%)	0 (0.0%)	0 (0.0%)
When I make a mistake, my parents/caregivers help me improve	0 (0.0%)	0 (0.0%)	0 (0.0%)
I am afraid of what will happen if I don’t listen to my parents/caregivers*	0 (0.0%)	0 (0.0%)	0 (0.0%)
My parents/caregivers treat me with respect	1 (0.0%)	0 (0.0%)	1 (0.6%)

*Reverse coded in both languages

^†^Reverse coded in English only

### Psychometric Testing of the Measure

While the average response on all survey questions combined can give a picture of a child’s overall subjective well-being, by creating subscales of survey items that are conceptually related to one another, researchers can more closely examine specific areas of children’s well-being. To this end, using data from *N =* 180 children in Kenya and Guatemala, the psychometric properties of the measure were evaluated by (1) conducting an exploratory factor analysis to create subscales in the measure, (2) evaluating the internal consistency of subscales with Cronbach’s alpha, and (3) evaluating the convergent validity of the subscales with their correlations with a unidimensional measure of life satisfaction.

### Sample and Participants

The measure was deployed in household surveys of all CTWWC participants in Kenya and Guatemala, the purpose of which was to evaluate CTWWC’s programming. Survey enumerators were given participants’ phone numbers and called them to arrange a home visit during which they administered the survey via face-to-face interview. If they could not be contacted, multiple attempts were made; ultimately, there were only 4 households (all in Kenya) that could not be reached via phone. Children were eligible to complete the child-informed well-being measure if they were between the ages of 11 to 18 and were receiving services from CTWWC (as they were either reunified with family after living in residential care, or were assessed to be at risk of entering residential care). In Kenya, there were 295 households eligible to participate in the household survey, and 89.2% (*N =* 263) did so (reasons for non-participation included relocation, inability to contact the family, and illness). Within these households, 55.3% of eligible children (i.e., children ages 11 to 18 receiving CTWWC case management) completed the child measures (*n =* 142); 29.6% of children (*n =* 76) could not be reached for surveying because they were away at boarding school, while some others no longer lived in the household. In Guatemala, 61 households were recruited to participate, and 96.7% did so (*n =* 59 participated and *n =* 2 declined to participate); the households contained 57 eligible children, of which 87.7% completed a child survey (*n =* 50 participated, while *n =* 3 children could not participate and *n =* 4 children no longer lived in the household at the time of the survey).

The measure included some questions that were designed to only be presented to children if they were enrolled in school (e.g., “I worry about having enough money for my education”), so we excluded children who were not in school from this analysis (*n =* 8 in Guatemala and *n =* 4 in Kenya). Thus, data from *n =* 138 children in Kenya and *n =* 42 children in Guatemala who were reunified with family after living in residential care or who were assessed to be at risk of entering residential care were used in our exploratory factor analysis.

### Quantitative Analysis

To determine how the overall tool could be divided into subscales, we undertook exploratory factor analysis (EFA). EFA is a method for uncovering the way in which latent variables (i.e., underlying concepts such as quality of life or relationship satisfaction) are related to observed variables (i.e., the responses to questions on a survey) was used to identify the factor structure of the scale (i.e., which survey items are statistically related to one another, and therefore can be grouped into subscales) (Watkins, 2018). The EFA approach was chosen because the researchers had no *a-priori* theory guiding, or expectation for, what sub-domains may have existed within the measure. The remainder of this section documents the statistical analyses and decisions made in conducting the EFA (for further reading, see Watkins, 2018).

Most items did not have any missing data; 17 out of the 43 items had between 1 and 4 missing cases (Table 4). For example, four responses were missing for *I get to play and have fun* (2.2% of the overall sample), and three from *I get along well with my friends* (1.7%). Little’s test of missing completely at random was χ2(42) = 57.1, *p* = .06. Since missingness in the sample was relatively low, and Little’s test was not statistically significant, missing data was handled via listwise deletion.

First, Bartlett’s test of sphericity (Bartlett, 1954) and the Kaiser Meyer Olkin (KMO) test were run in order to determine whether the data were appropriate for EFA. Second, in order to determine the optimal number of factors, the researcher visually examined scree plots and noted how many eigenvalues were greater than 1. The EFA was based on polychoric correlations (as the items are ordinal with fewer than five response options), used an iterated principal axis factor extraction (which is better suited for small sample sizes than maximum likelihood estimation), and used oblique (*promax*) rotation (Watkins, 2018).

In order to make decisions regarding which items should be included in each subscale, we followed the “simple structure” concept, which suggests that “(a) each factor should be saliently loaded by at least three variables (i.e., overdetermined), (b) each variable should load saliently on only one factor (no complex or cross-loadings), (c) each factor should demonstrate internal consistency reliability ≥ .70, and (d) all factors should be theoretically meaningful” (Watkins, 2018, pp. 234–235). A variable was considered saliently loaded on a factor if its factor loading (i.e., the strength of the relationship between the variable and the factor) was 0.40 or greater, and internal consistency (i.e., how closely survey items within one factor are related to one another) was assessed by calculating Cronbach’s alpha. Thus, if an item’s loading was less than 0.40, it was a candidate for deletion.

Finally, the measure’s convergent validity (how well it relates to another measure that is supposed to assess a similar construct) was gauged by examining the factors’ correlations with the unidimensional measure Overall Life Satisfaction (OLS), in which respondents were asked to rate how happy or satisfied they were with their life overall on a scale of 0 to 10, where 0 represented not at all satisfied and 10 completely satisfied. A visual aid was provided to help respondents understand the scale. The wording of this question, which is originally from Campbell (1976), is now widely used in the Personal Well-being Index – School Children (Cummins & Lau, 2005), and this question is used with a similar visual aid in the Children’s Worlds International Survey of Children’s Well-Being (Children’s Worlds, n.d.). A study with Serbian adolescents found that it had adequate criterion validity and good convergent validity with depression, anxiety, stress, and negative affect, and positive affect (Jovanović, 2016). Besides the OLS and the measure described in this paper, participants also completed brief measures of family and community acceptance, which were not included in the aim of the current study.

## Results

The mean age of participants in the complete *N* = 180 dataset was 14.3 years (*SD =* 2.0), and almost half (44.4%) were girls. Most participants (87.2%) had been reunified with family after living in residential care, while the others were identified as being at risk of entering residential care. About a third (36.1%) of participants were cared for by someone other than their biological parents, and this was more common in Kenya than Guatemala. After listwise deletion, 164 participants remained for analysis (91.1%); their demographic characteristics are listed in Table 5.

**Table 5:** Exploratory factor analysis dataset sample characteristics (n [%] or M [SD])

	Kenya (*n =* 130)	Guatemala (*n =* 34)	Overall (*n* = 164)
Case type			
At-risk	14 (10.8%)	7 (20.6%)	21 (12.8%)
Reunified	116 (89.2%)	27 (79.4%)	143 (87.2%)
Female	57 (43.9%)	15 (44.1%)	72 (43.9%)
Living arrangements			
Both biological parents	12 (9.2%)	16 (47.1%)	28 (17.1%)
One biological parent	64 (49.2%)	14 (41.2%)	78 (47.6%)
Neither biological parent	54 (41.5%)	4 (11.8%)	58 (35.4%)
Mean age (years)	14.1 (2.0)	14.9 (2.0)	14.2 (2.0)

Bartlett’s test of sphericity was χ2(903) = 3086.791, *p* < .001 (Bartlett, 1954), and the Kaiser Meyer Olkin (KMO) statistic was 0.833 (Kaiser, 1974), indicating that the data were appropriate for exploratory factor analysis (Bartlett’s test should be statistically significant in order to conduct EFA, while a KMO of 0.833 is considered “meritorious” according to Kaiser). Visual analysis of scree plots suggested between 3 and 5 factors should be retained, while 11 eigenvalues were greater than 1, and Horn’s parallel analysis of factors suggested 5 factors be retained. Thus, factor structures with six, five, four, and three factors were sequentially examined. Solutions with five and six factors resulted in multiple factors that had only one or two items saliently loaded onto the factor. The four-factor solution was inadequate, with four cross-loaded items (i.e., items loaded onto more than one factor), and with the fourth factor having an internal consistency of α < 0.60; the four factors also were determined to be insufficiently distinct in terms of subject matter.

The three-factor solution was judged to be adequate, with factors covering distinct content areas and having only two cross-loadings. All loadings from this solution are displayed in Table 6. We named factor 1 *care and safety* (12 items), factor 2 *basic needs* (13 items), and factor 3 *leisure and freedom* (7 items). Using the “simple structure” criteria (Watkins, 2018), we removed seven items that did not load saliently onto any factor (*I like my teachers at school; I’m treated differently from other children in my community; I’m treated differently from the other children in my household; If I needed something that my parents/caregivers can’t provide, there are others who would help; I would be given medicine if I needed it; I have enough time to study; I am afraid of what will happen if I don’t listen to my parents/caregivers*). Then, three items were removed because it was determined that they did not match the theoretical meaning of the factors onto which they loaded (*I am as happy as other kids my age; My health is good; My teachers treat me with respect*). Two items loaded saliently onto more than one factor (*At home, I have everything I need to keep myself clean; I feel I am supported by my relatives*), but because the item about keeping clean was theoretically relevant to the rest of the *basic needs* factor, it was retained on factor 2 despite the cross-loading; as the item about support from relatives was related to both basic needs as well as care and safety, this item was dropped. This final scale is included as Table 7.

**Table 6:** Results of exploratory factor analysis

Item	Loadings onto each factor			Decision
	Factor 1: Care & safety	Factor 2: Basic needs	Factor 3: Leisure & freedom	
When I make a mistake, my parents/caregivers help me improve	**0.84**	0.07	− 0.16	Retained as factor 1
I have someone to turn to for advice and guidance	**0.83**	− 0.10	0.01	Retained as factor 1
I have people I can talk to when I have a problem	**0.82**	0.13	− 0.05	Retained as factor 1
My parents/caregivers treat me with respect	**0.78**	− 0.18	0.15	Retained as factor 1
I get love and care from my parents/caregivers	**0.76**	0.15	0.02	Retained as factor 1
I have adults in my life who understand me	**0.68**	0.00	0.21	Retained as factor 1
The adults in my life teach me how to be successful in the future	**0.67**	0.03	0.08	Retained as factor 1
I feel like I’m part of my family	**0.64**	0.33	− 0.01	Retained as factor 1
If I want something, my parents/caregivers will listen and consider it	**0.62**	0.09	− 0.03	Retained as factor 1
I have someone to ask for help if I feel unsafe	**0.62**	0.29	0.07	Retained as factor 1
Someone would take me to the hospital/clinic/doctor if I needed it	**0.57**	0.06	0.06	Retained as factor 1
My home is peaceful	**0.50**	0.28	0.11	Retained as factor 1
*I like my teachers at school*	0.36	− 0.21	0.27	Dropped as loadings < 0.40
*I’m treated differently from [Spanish: the same as] other children in my village/neighborhood/compound/community*	0.34	− 0.13	0.29	Dropped as loadings < 0.40
*If I needed something that my parents/caregivers can’t provide, there are others who would help*	0.31	0.29	− 0.19	Dropped as loadings < 0.40
*I would be given medicine if I needed it*	0.29	0.28	0.24	Dropped as loadings < 0.40
I like the food I eat	0.01	**0.83**	− 0.07	Retained as factor 2
My home has a good environment for studying	0.06	**0.75**	0.05	Retained as factor 2
I have a comfortable place to sleep at night	− 0.19	**0.74**	0.22	Retained as factor 2
I can eat until I am satisfied	0.03	**0.71**	0.09	Retained as factor 2
My diet is well-balanced and nutritious	0.12	**0.70**	0.05	Retained as factor 2
I worry about having enough money for my education	− 0.22	**0.68**	0.06	Retained as factor 2
I can choose what to eat and when	0.26	**0.66**	− 0.32	Retained as factor 2
I feel safe where I live	0.10	**0.61**	0.32	Retained as factor 2
I eat at least two meals a day	0.17	**0.58**	0.02	Retained as factor 2
I am happy with my clothing and shoes	0.29	**0.56**	− 0.02	Retained as factor 2
At home, I have everything I need to keep myself clean	0.44	**0.53**	− 0.20	Retained as factor 2
I have the materials I need for school	0.31	**0.45**	− 0.01	Retained as factor 2
*I am as happy as other kids my age*	0.24	0.44	0.25	Dropped as not theoretically matched to factor
*I feel I am supported by my relatives*	0.42	0.44	− 0.08	Dropped due to cross-loading
My work or chores impact my ability to do well in school	− 0.29	**0.40**	0.19	Retained as factor 2
*I have enough time to study*	0.19	0.39	0.29	Dropped as loadings < 0.40
*I am afraid of what will happen if I don’t listen to my parents/caregivers*	− 0.26	0.35	− 0.02	Dropped as loadings < 0.40
I have fun with my friends	− 0.06	− 0.18	**0.75**	Retained as factor 3
I get along well with my friends	0.02	0.04	**0.73**	Retained as factor 3
*My health is good*	− 0.13	0.15	0.72	Dropped as not theoretically matched to factor
I am happy with how many friends I have	0.15	− 0.01	**0.72**	Retained as factor 3
I get to play and have fun	0.11	0.17	**0.59**	Retained as factor 3
I get to pursue my hobbies and interests	0.00	0.39	**0.52**	Retained as factor 3
I have enough time to rest and sleep	0.09	0.28	**0.52**	Retained as factor 3
I have freedom to go out	− 0.12	0.14	**0.50**	Retained as factor 3
*My teachers treat me with respect*	0.31	0.00	0.44	Dropped as not theoretically matched to factor
*I’m treated differently from [Spanish: the same as] the other children in my household*	0.33	− 0.35	0.39	Dropped as loadings < 0.40

**Note** Bolded statistics indicate items retained on their respective factors; italics indicate dropped items.

**Table 7:** Final scale in English and Spanish

English survey	Spanish item
*I am now going to read you some statements. I would like you to please tell me how often each statement is true for you: (0) None of the time, (1) Some of the time, or (2) All of the time. If you would like me to repeat the statements at any time, please stop me and ask me to repeat. Do you understand? (See if child has any questions.) May I begin?*	*Ahora les voy a leer algunas frases. Me gustaría que me dijera que tan frecuente la frase sea cierta para usted: (0) Ninguna de las veces, (1) Algunas veces o (2) Siempre. Si desea que repita las frase, por favor dimelo y con gusto lo hare. ¿Entiendes? (Si el niño tiene alguna pregunta, respóndasela de la manera más fácil y sencilla). ¿Puedo empezar?*
Care and safety subscale	
When I make a mistake, my parents/caregivers* help me improve	Cuando cometo un error, mis padres/cuidadores me ayudan a mejorar
I have someone to turn to for advice and guidance	Tengo a alguien a quien acudir para pedir consejo y orientación
I have people I can talk to when I have a problem	Tengo personas con quien puedo hablar cuando tengo un problema
My parents/caregivers treat me with respect	Mis padres/cuidadores me tratan con respeto
I get love and care from my parents/caregivers	Recibo amor y cuidado de mis padres/cuidadores
I have adults in my life who understand me	Tengo adultos en mi vida que me comprenden
The adults in my life teach me how to be successful in the future	Los adultos en mi vida me enseñan cómo tener éxito en el futuro
I feel like I’m part of my family	Me siento parte de mi familia
If I want something, my parents/caregivers will listen and consider it	Si quisiera algo, mis padres / cuidadores me escucharan y consideraran dármelo
I have someone to ask for help if I feel unsafe	Tengo a quien pedir ayuda si me siento en peligro
Someone would take me to the hospital/clinic/doctor if I needed it	Si lo necesito, alguien me lleva al médico / clínica / puesto de salud u hospital
My home* is peaceful	Mi hogar es tranquilo
Basic needs subscale	
I like the food I eat	Me gusta la comida que como
My home has a good environment for studying†	Mi casa tiene un buen ambiente para estudiar†
I have a comfortable place to sleep at night	Tengo un lugar cómodo para dormir por la noche
I can eat until I am satisfied	Puedo comer hasta quedar lleno
My diet is well-balanced and nutritious	Mi dieta es balanceada y nutritiva
I worry about having enough money for my education (R)†	Me preocupa no tener suficiente dinero para pagar mis gastos de educación (R)†
I can choose what to eat and when	Puedo elegir qué y cuándo comer
I feel safe where I live	Me siento seguro donde vivo
I eat at least two meals a day	Yo como por lo menos dos comidas al día
I am happy with my clothing and shoes	Estoy feliz con mi ropa y zapatos
At home, I have everything I need to keep myself clean	En casa tengo todo lo que necesito para mantenerme limpio
I have the materials I need for school†	Tengo los materiales que necesito para la escuela†
My work or chores impact my ability to do well in school (R)†	Mis oficios del hogar o mi trabajo afectan mi rendimiento en la escuela (R)†
*Leisure and freedom subscale*	
I have fun with my friends	Me divierto con mis amigos
I get along well with my friends	Me llevo bien con mis amigos
I am happy with how many friends I have	Estoy contento con la cantidad de amigos que tengo
I get to play and have fun	Puedo jugar y divertirme
I get to pursue my hobbies and interests	Puedo dedicarme a mis pasatiempos e intereses
I have enough time to rest and sleep	Tengo suficiente tiempo para descansar y dormir
I have freedom to go out	Tengo la libertad de salir fuera de la casa

(R) designates reverse-coded items.

* The terms “parent/caregiver” and “home” can be replaced so as to be most applicable to the child’s current living situation (e.g., my caregiver, my auntie, my home, the children’s home).

† These items should only be presented to children enrolled in school.

The internal consistency of the *care and safety* factor was α = 0.88, *basic needs* was α = 0.85, and *leisure and freedom* was α = 0.72, and all items improved the alpha values of their respective scales, indicating that each factor consisted of survey items that were related to one another.

As a sensitivity analysis, we evaluated the factor loadings onto a three-factor solution using only data from Kenya, since Kenya had the larger sample size. The items that loaded < 0.40 onto the three factors results were similar, but not exactly the same, to those that used both countries’ samples pooled together. For factor 1, there was 72.1% match in loaded items between the Kenya-only and the pooled sample. For factor 2, the match was 81.4%, and for factor 3, the match was 88.4%. We ultimately decided that in the pooled sample solution, the items in each factor were more thematically similar to one another, and there was a better balance in the number of items per factor, compared to the Kenya-only solution.

As another sensitivity analysis, another three-factor exploratory factor analysis was run for the pooled sample with the dropped items excluded. The same items loaded saliently onto the same factors as in the original solution. The proportion of variance of all these items explained by the *care and safety* factor was 33.2%, by *basic needs* was 31.5%, and by *leisure and freedom* was 19.4%.

Finally, scale scores were created of each of the three factors by calculating the mean of their items. To examine convergent validity, the correlations between the Overall Life Satisfaction measure and factor were examined; it was significantly correlated with the *care and safety* scale (*r =* .42, *p < .*001), *basic needs* (*r = .*51, *p <* .001), and *leisure and freedom* (*r* = .23, *p = .*002), indicating good convergent validity of all three factors.

## Discussion

The utility of well-being measures that are tailored to the needs and priorities of diverse populations is well recognized (McGregor et al., 2015; Sharma et al., 2013). Although research about children’s development and well-being can be critical in shaping policy and practice around the use of residential care institutions for children (Goldman et al., 2020; van IJzendoorn et al., 2020), and although qualitative research suggests that the salient experiences of children in residential care are unique (Roche, 2019), we could not identify any measures of well-being specifically tailored to the priorities of children who have experienced residential care. This study contributes to filling this gap by constructing survey questions about children’s subjective well-being based on qualitative analysis of focus groups with children and young people who have lived in RCIs in Kenya and Guatemala, resulting in a 43-item child-informed measure that is specifically designed for use with children in RCIs, who have left RCIs, who are at risk of entering RCIs, and any potential comparison groups. Exploratory factor analysis suggested that the measure assessed three underlying constructs measured by 32 items, which we call *care and safety, basic needs*, and *leisure and freedom.* This exploratory factor analysis enhanced the utility of the measure because it suggested three distinct subscales that can be calculated to more finely assess specific dimensions of children’s subjective well-being.

A strength of this scale is that it relies on child self-report about their subjective well-being. Much existing research on children in non-parental care looks at children’s objective well-being (e.g., health status, educational attainment, indicators of wealth and poverty), and this research often informs policymaking. For example, the Government of Ghana explicitly cites “[e]vidence from child development literature and neuroscience” as justification for transitioning children out of residential care and into families (Ghana Department of Social Welfare & UNICEF, 2020, p. 25); such evidence is largely based on objective measures of child well-being. However, in line with the Convention on the Rights of the Child’s emphasis on child participation, data collection efforts that inform policymaking should include the voice and subjective experiences of affected children themselves (United Nations General Assembly, 1989), and we hope that this measure can foster the collection and use of such data from children. For example, if deinstitutionalization programs were monitored using data collected via our measure, governments would be able to use children’s perspectives to empirically assess whether their program was succeeding or needed strengthening in, for example, provision of material assistance to reunified families, or greater social worker oversight of children’s safety.

Analysis of data from the focus groups reinforce the literature’s findings that children in RCIs care about the level of agency and decision-making power they have over their own lives (Roche, 2019). This theme of autonomy, and ability to decide how to spend one’s time, freedom of movement, and freedom to play and spend time with friends, was important to young people, yet seems to be relatively unexplored in quantitative research. Indeed, although the Convention on the Rights of the Child recognizes “the right of the child to rest and leisure, to engage in play and recreational activities” (United Nations General Assembly, 1989, art. 31), none of the quantitative studies of children in RCIs that we reviewed mentioned leisure or play as an aspect of child well-being (e.g., van IJzendoorn et al., 2020, James et al., 2017, Gray et al., 2015; Whetten et al., 2009). Thus, one of the current study’s significant contributions is the construction and validation of a quantitative subscale that assesses children’s subjective perspective on leisure and freedom in their lives.

Prior literature on children’s well-being in RCIs also confirms the importance of having their basic, material needs met. Poverty is a significant driver of children entering residential care in many contexts, and caregivers may choose to place children in RCIs so that they can access food, healthcare, and education (van IJzendoorn et al., 2020). One study in Ghana found that children in RCIs had better access to material resources than children who had been reunified with family after living in RCIs (James et al., 2017). Thus, it is important that research and evaluation assess child poverty, food security, access to education, and other dimensions of basic needs, in order to inform policies around the use of RCI and support services that may need to be provided to children’s families after family reunification. While other measures can provide more robust, objective measures of household economic status, such as those employed in the Demographic and Health Surveys (Staveteig & Mallick, 2014), the “basic needs” subscale of our measure can provide an important complementary perspective, which is children’s own perceptions on whether their material needs are being met in areas they prioritize. For instance, research on child well-being in the UK suggests that child depression is more closely linked to subjective measures like perceived financial strain, and feelings of not having the same material possessions as their peers, than it is with objective measures of poverty based on income (The Children’s Society, 2019).

For children who have lived in RCIs or who are at risk of entering RCIs, their experience of being parented and receiving care is particularly important. Children may enter RCIs due to abuse or neglect or the presence of violence or dysfunction in their family. At the same time, literature suggests that abuse, violence, neglect, and lack of individualized attention, can also characterize RCIs across low- and middle-income countries (Dozier et al., 2012; Rus et al., 2017). A study of five low- and middle-income countries found that over half of children who had lost a parent or were separated from their parents had experienced physical or sexual abuse by age 13, and this was true whether they lived in RCIs or families; 31% of children in RCIs had experienced violence in their RCI and 37% of children in families had experienced violence in the family home (Gray et al., 2015). Other participatory studies have also found young people who have lived in non-parental care frequently mention the importance of “love” with great consistency across different regions of the world (Butler et al., 2021; Independent Care Review, 2020). Thus, the “care and safety” subscale of this measure is a tool for assessing whether children feel they are receiving love and care, receiving the parenting or caregiving they need, and whether they feel safe in their homes. Obtaining this information from children themselves, rather than relying only on caregivers’ or other adults’ reports, is critical.

### Limitations

One limitation of this study is that only one person conducted the rapid coding of the focus group data, with no second coder to enhance the reliability and validity of the procedures; however, the member checking process with participants mitigated some of this potential source of bias. The psychometric analysis in this study was also limited by its small sample size, which precluded our ability to do a split sample analysis (i.e., conducting exploratory factor analysis on one half of the sample and assessing the fit of the best-fitting model with confirmatory factor analysis in the other). Another limitation is that an item that was developed from the focus group data, “I am happy with how much time I get to spend with my family,” was not used in Kenya due to a survey programming error, despite this item being relevant to the population’s subjective well-being. Although the study aimed to recruit all children ages 11 to 18 who were enrolled in CTWWC’s programming, children who were not enrolled in school were excluded from this analysis, and many children in Kenya could not be surveyed because they were away at boarding school; these could have introduced bias to the sample. In addition, since two items were not reverse-coded in Spanish, there could have been measurement differences between the two languages. Finally, and crucially, this study only used data from only two countries, Kenya and Guatemala, and repeating this process in more contexts could enhance its applicability worldwide. Without these limitations, it is possible that the EFA would have resulted in different factor loadings.

### Implications and Future Directions

This study adopted an innovative approach to the measurement of outcomes for children who had spent time in residential care, using participatory, qualitative methods with children and young people to inductively construct a new quantitative measure. The results of this study integrate child participation in multiple layers: as this is a child-report tool, the *answers* children give to this survey embody child participation, and the tool was designed via participatory methods, the *questions* asked of children also represent children’s priorities.

It should be noted that there is a tension in developing measures contextualized for diverse populations: that is, how specific is too specific, and how general is too general? If a measure is too finely tailored to a specific group, its applicability can be impractically narrow. If a measure is developed to be used too broadly, then it can fail to measure nuances that are important to the population of interest. Recognizing that much of the influential discourse on children’s care happens at a global level (e.g., the UN Committee on the Rights of the Child 2021 Day of General Discussion on children’s rights and alternative care [Office of the High Commissioner for Human Rights, 2021], the Guidelines for the Alternative Care of Children [United Nations General Assembly, 2010], etc.), we aimed to create a measure that can be useful for research across various low- and middle-income countries by pooling the common experiences of participants in the very different countries of Kenya and Guatemala. For example, a multi-country data analysis of our measure that compares children in residential care and foster care could inform UNICEF practice guidelines around alternative family care, or the allocation of resources to strengthen alternative care in various regions of the world. However, researchers, practitioners, and policymakers may disagree about what balance between specificity and generalizability is ideal for measures used to inform policymaking. This question cuts to the core of global research, and those working in global development and human rights must elevate and grapple with this issue in future research.

Research on children’s care that aims to be highly localized may find it more useful to have a measure specifically tailored to their particular cultural context, because the experiences of alternative care, and residential care more specifically, can differ greatly from country to country. In these cases, this study offers useful contributions as well, as it can either provide a replicable methodology that another researcher could use to construct their own child-informed, contextual measure of child well-being from their own focus groups, or they could take these questions or subscales as a starting point for further cultural adaptation and contextualization.

It is critical that both qualitative and quantitative research on children’s care uplifts the perspectives of children and young people with the lived experience of alternative care that is residential in nature. This study provides a useful tool and example methodology for embedding child participation in research, ensuring that when such research influences policy making on the use of residential models as part of the child welfare system in low- and middle-income countries, children’s perspectives are at the table.

## References

[CR1] Bartlett, M. S. (1954). A note on the multiplying factors for various χ ^2^ approximations. *Journal of the Royal Statistical Society: Series B (Methodological)*, *16*(2), 296–298. 10.1111/j.2517-6161.1954.tb00174.x.

[CR2] Betancourt, T. S., Fawzi, M. K. S., Bruderlein, C., Desmond, C., & Kim, J. Y. (2010). Children affected by HIV/AIDS: SAFE, a model for promoting their security, health, and development. *Psychology Health & Medicine*, *15*(3), 243–265. 10.1080/13548501003623997. 10.1080/13548501003623997 20480431

[CR3] Butler, K., Currie, V., Reid, K., & Wright, L. (2021). *Make our voices count: Children and young peoples’ responses to a global survey for the Day of General Discussion 2021 on Children’s Rights and Alternative Care*. https://www.ohchr.org/en/events/days-general-discussion-dgd/2021/2021-day-general-discussion-childrens-rights-and.

[CR4] Campbell, A., Rodgers, W. L., & Converse, P. E. (1976). *The quality of American life: Perceptions, evaluations, and satisfactions*. Russell Sage Foundation.

[CR5] Catholic Relief Services (n.d.). *Changing the Way We Care*. https://www.changingthewaywecare.org/.

[CR6] Changing the Way We Care (2020). *Embracing childhood: Opinion study on residential care and alternative family care in Guatemala*. https://bettercarenetwork.org/sites/default/files/2020-11/28_EN_GT%20Opinion%20Study%20CTWWC.pdf.

[CR7] Chege, N., & Ucembe, S. (2020). Kenya’s over-reliance on institutionalization as a child care and child protection model: A root-cause approach. *Social Sciences*, *9*(4), 57. 10.3390/socsci9040057.

[CR8] Cheney, K. E., & Rotabi, K. S. (2014). Addicted to orphans: How the global orphan industrial complex jeopardizes local child protection systems. In C. Harker, K. Hörschelmann, & T. Skelton (Eds.), *Conflict, Violence and Peace* (pp. 1–19). Springer. 10.1007/978-981-4585-98-9_3-1.

[CR9] Children’s Worlds (n.d.). *Children’s Worlds International Survey of Children’s Well-Being 8 years-old questionnaire*. https://isciweb.org/the-questionnaire/using-the-questionnaires/.

[CR10] Collins, D. (2003). Pretesting survey instruments: An overview of cognitive methods. *Quality of Life Research*, *12*(3), 229–238. 10.1023/A:1023254226592. 12769135 10.1023/a:1023254226592

[CR11] Creswell, J. W., & Poth, C. N. (2018). *Qualitative inquiry & research design: Choosing among five approaches*. SAGE. Fourth edition.

[CR12] Cummins, R. A., & Lau, A. L. D. (2005). *Personal Wellbeing Index – School Children (PWI-SC)*. http://www.acqol.com.au/uploads/pwi-sc/pwi-sc-english.pdf.

[CR24] del Manzo Chávez, M. (2021). C. Emotional psychological impact of institutionalization on children and early adolescents. In B. E. Barcelata Eguiarte & P. Suárez Brito (Eds.), *Child and adolescent development in risky adverse contexts* (pp. 223–240). Springer. 10.1007/978-3-030-83700-6_11.

[CR13] Desmond, C., Watt, K., Saha, A., Huang, J., & Lu, C. (2020). Prevalence and number of children living in institutional care: Global, regional, and country estimates. *The Lancet Child & Adolescent Health*, *4*(5), 370–377. 10.1016/S2352-4642(20)30022-5. 32151317 10.1016/S2352-4642(20)30022-5

[CR14] Dozier, M., Zeanah, C. H., Wallin, A. R., & Shauffer, C. (2012). Institutional care for young children: Review of literature and policy implications. *Social Issues and Policy Review*, *6*(1), 1–25. 10.1111/j.1751-2409.2011.01033.x. 23513085 10.1111/j.1751-2409.2011.01033.x PMC3600163

[CR15] Ghana Department of Social Welfare & UNICEF (2020). Guidelines for deinstitutionalisation of residential homes for children: Transitioning to family-based care in Ghana. https://www.unicef.org/ghana/reports/guidelines-deinstitutionalisation-residential-homes-children.

[CR16] Goldman, P. S., Bakermans-Kranenburg, M. J., Bradford, B., Christopoulos, A., Ken, P. L. A., Cuthbert, C., Duchinsky, R., Fox, N. A., Grigoras, S., Gunnar, M. R., Ibrahim, R. W., Johnson, D., Kusumaningrum, S., Agastya, N. L. P. M., Mwangangi, F. M., Nelson, C. A., Ott, E. M., Reijman, S., van IJzendoorn, M. H., & Sonuga-Barke, E. J. S. (2020). Institutionalisation and deinstitutionalisation of children 2: Policy and practice recommendations for global, national, and local actors. *The Lancet Child & Adolescent Health*, 2352464220300602. 10.1016/S2352-4642(20)30060-2. 10.1016/S2352-4642(20)30060-2 PMC7311356 32589873

[CR17] Gray, C. L., Pence, B. W., Ostermann, J., Whetten, R. A., O’Donnell, K., Thielman, N. M., & Whetten, K. (2015). Prevalence and incidence of traumatic experiences among orphans in institutional and family-based settings in 5 low- and middle-income countries: A longitudinal study. *Global Health: Science and Practice*, *3*(3), 395–404. 10.9745/GHSP-D-15-00093. 26374801 10.9745/GHSP-D-15-00093 PMC4570014

[CR18] Independent Care Review (2020). *The promise*. https://www.carereview.scot/conclusions/independent-care-review-reports/.

[CR19] James, S. L., Roby, J. L., Powell, L. J., Teuscher, B. A., Hamstead, K. L., & Shafer, K. (2017). Does family reunification from residential care facilities serve children’s best interest? A propensity-score matching approach in Ghana. *Children and Youth Services Review*, *83*, 232–241. 10.1016/j.childyouth.2017.10.032.

[CR20] Jovanović, V. (2016). The validity of the satisfaction with Life Scale in adolescents and a comparison with single-item life satisfaction measures: A preliminary study. *Quality of Life Research*, *25*(12), 3173–3180. 10.1007/s11136-016-1331-5. 27262574 10.1007/s11136-016-1331-5

[CR21] Kaiser, H. F. (1974). An index of factorial simplicity. *Psychometrika*, *39*(1), 31–36. 10.1007/BF02291575.

[CR22] Kenya Department of Children’s Services (2020). *Situational analysis report for children’s institutions in five counties: Kiambu, Kilifi, Kisumu, Murang’a and Nyamira summary report*. https://bettercarenetwork.org/sites/default/files/2021-03/18.21_SitAn%20Summary.pdf.

[CR23] Kirk, A. R., Groark, C. J., & McCall, R. B. (2017). Institutional care environments for infants and young children in Latin America and the Caribbean. In A. V. Rus, S. R. Parris, & E. Stativa (Eds.), *Child maltreatment in residential care* (pp. 401–418). Springer. 10.1007/978-3-319-57990-0_19.

[CR25] Martin, F. S., & Zulaika, G. (2016). Who cares for children? A descriptive study of care-related data available through global household surveys and how these could be better mined to inform policies and services to strengthen family care. *Global Social Welfare*, *3*(2), 51–74. 10.1007/s40609-016-0060-6.

[CR26] McGregor, A., Coulthard, S., & Camfield, L. (2015). *The role of well-being methods in development policy and practice* (No. 4; Development Progress Project Note). ODI. https://www.odi.org/publications/9657-measuring-what-matters-role-well-being-methods-development-policy-and-practice.

[CR27] Ng, L. C., Kanyanganzi, F., Munyanah, M., Mushashi, C., & Betancourt, T. S. (2014). Developing and validating the Youth Conduct problems Scale-Rwanda: A mixed methods approach. *Plos One*, *9*(6), e100549. 10.1371/journal.pone.0100549. 24949628 10.1371/journal.pone.0100549 PMC4065113

[CR28] OECD (2011). *Compendium of OECD well-being indicators*. *OECD*. https://www.oecd.org/general/compendiumofoecdwell-beingindicators.htm.

[CR29] OECD. (2013). OECD guidelines on measuring subjective well-being. *OECD*. 10.1787/9789264191655-en. 24600748

[CR30] Office of the High Commissioner for Human Rights (2021). 2021 day of general discussion: Children’s rights and alternative care. https://www.ohchr.org/en/events/days-general-discussion-dgd/2021/2021-day-general-discussion-childrens-rights-and.

[CR31] Oxfam Scotland (2013). *Oxfam Humankind Index: The new measure of Scotland’s prosperity, second results*. Oxfam GB. https://policy-practice.oxfam.org/resources/oxfam-humankind-index-the-new-measure-of-scotlands-prosperity-second-results-293743/.

[CR32] Pandya, S. P. (2018). Spirituality for wellbeing of bereaved children in residential care: Insights for spiritually sensitive child-centred social work across country contexts. *Child & Adolescent Social Work Journal*, *35*, 181–195. 10.1007/s10560-0170509-1.

[CR33] Roche, S. (2019). A scoping review of children’s experiences of residential care settings in the global South. *Children and Youth Services Review*, *105*, 104448. 10.1016/j.childyouth.2019.104448.

[CR34] Rus, A. V., Parris, S. R., Stativa, E., Bejenaru, A., Webster, D., Wente, R., J., & Cojocaru, S. (2017). An introduction to maltreatment of institutionalized children. In A. V. Rus, S. R. Parris, & E. Stativa (Eds.), *Child maltreatment in residential care* (pp. 1–25). Springer International Publishing. 10.1007/978-3-319-57990-0_1.

[CR35] Saldaña, J. (2016). *The coding manual for qualitative researchers* (Vol. 3(E). SAGE. [Third edition]).

[CR36] Salifu Yendork, J. S., & Somhlaba, N. Z. (2014). Stress, coping and quality of life: An exploratory study of the psychological wellbeing of Ghanaian orphans placed in orphanages. *Children & Youth Services Review*, *46*, 28–37. https://doi.org/10.1016/j. childyouth.2014.07.0258.

[CR37] Selwyn, J., Wood, M., & Newman, T. (2017). Looked after children and young people in England: Developing measures of subjective well-being. *Child Indicators Research*, *10*(2), 363–380. 10.1007/s12187-016-9375-1.

[CR38] Senefeld, S., Strasser, S., Campbell, J., & Perrin, P. (2011). Measuring adolescent well-being: The development of a standardized measure for adolescents participating in orphans and vulnerable children programming. *Vulnerable Children and Youth Studies*, *6*(4), 346–359. 10.1080/17450128.2011.635722.

[CR39] Sharma, D. K. B., Lopez, E. D. S., Mekiana, D., Ctibor, A., & Church, C. (2013). What makes life good? Developing a culturally grounded quality of life measure for Alaska Native college students. *International Journal of Circumpolar Health*, *72*(1), 21180. 10.3402/ijch.v72i0.21180. 10.3402/ijch.v72i0.21180 PMC3753161 23984302

[CR40] Staveteig, S., & Mallick, L. (2014). *Intertemporal comparisons of poverty and wealth with DHS data: A harmonized asset index approach* (No. 15; DHS Methodological Reports). ICF International. https://dhsprogram.com/pubs/pdf/MR15/MR15.pdf.

[CR41] The Children’s Society (2019). *The good childhood report: 2019 summary*. https://childhub.org/en/child-protection-online-library/good-childhood-report-2019.

[CR42] Tomyn, A. J., & Cummins, R. A. (2011). The subjective wellbeing of high-school students: Validating the personal wellbeing index—school children. *Social Indicators Research*, *101*(3), 405–418. 10.1007/s11205-010-9668-6.

[CR43] United Nations General Assembly (1989). *Convention on the rights of the child*. https://www.ohchr.org/EN/ProfessionalInterest/Pages/CRC.aspx.

[CR44] United Nations General Assembly (2010). *Guidelines for the alternative care of children*. https://digitallibrary.un.org/record/673583.

[CR45] van IJzendoorn, M. H., Bakermans-Kranenburg, M. J., Duschinsky, R., Fox, N. A., Goldman, P. S., Gunnar, M. R., Johnson, D. E., Nelson, C. A., Reijman, S., Skinner, G. C. M., Zeanah, C. H., & Sonuga-Barke, E. J. S. (2020). Institutionalisation and deinstitutionalisation of children 1: A systematic and integrative review of evidence regarding effects on development. *The Lancet Psychiatry*, S2215036619303992. 10.1016/S2215-0366(19)30399-2. 10.1016/S2215-0366(19)30399-2 32589867

[CR46] Voukelatou, V., Gabrielli, L., Miliou, I., Cresci, S., Sharma, R., Tesconi, M., & Pappalardo, L. (2021). Measuring objective and subjective well-being: Dimensions and data sources. *International Journal of Data Science and Analytics*, *11*(4), 279–309. 10.1007/s41060-020-00224-2.

[CR47] Walker, P., Michaelson, J., & Trebeck, K. (2012). *Oxfam Humankind Index for Scotland—Background* (Oxfam Research Report). Oxfam. https://www.northernstarassociates.co.uk/wp-content/uploads/2013/02/HKIcmrApril2012.pdf.

[CR48] Watkins, M. W. (2018). Exploratory factor analysis: A guide to best practice. *Journal of Black Psychology*, *44*(3), 219–246. 10.1177/0095798418771807.

[CR50] Whetten, K., Ostermann, J., Whetten, R. A., Pence, B. W., O’Donnell, K., Messer, L. C., Thielman, N. M., & The Positive Outcomes for Orphans (POFO) Research Team. (2009). A comparison of the wellbeing of orphans and abandoned children ages 6–12 in institutional and community-based care settings in 5 less wealthy nations. *Plos One*, *4*(12), e8169. 10.1371/journal.pone.0008169. 20020037 10.1371/journal.pone.0008169 PMC2790618

[CR49] Whetten, K., Ostermann, J., Pence, B. W., Whetten, R. A., Messer, L. C., Ariely, S., O’Donnell, K., Wasonga, A. I., Vann, V., Itemba, D., Eticha, M., Madan, I., Thielman, N. M., & The Positive Outcomes for Orphans (POFO) Research Team. (2014). Three-year change in the wellbeing of orphaned and separated children in institutional and family-based care settings in five low- and middle-income countries. *Plos One*, *9*(8), e104872. 10.1371/journal.pone.0104872. 25162410 10.1371/journal.pone.0104872 PMC4146542

[CR51] Wood, M., & Selwyn, J. (2017). Looked after children and young people’s views on what matters to their subjective well-being. *Adoption & Fostering*, *41*(1), 20–34. 10.1177/0308575916686034.

[CR52] World Health Organization. (1998). *The World Health Organization Quality of Life (WHOQOL) user manual*. World Health Organization. https://www.who.int/mental_health/publications/whoqol/en/.

[CR53] Wright, A. W., Richard, S., Sosnowski, D. W., & Kliewer, W. (2019). Predictors of better functioning among institutionalized youth: A systematic review. *Journal of Child and Family Studies*. 10.1007/s10826-019-01527-0.

[CR54] Zhang, M. F., & Selwyn, J. (2020). The subjective well-being of children and young people in out of home care: Psychometric analyses of the your life, your care survey. *Child Indicators Research*, *13*(5), 1549–1572. 10.1007/s12187-019-09658-y.

